# Wheelchair accessibility to public buildings in the Kumasi metropolis, Ghana

**DOI:** 10.4102/ajod.v6i0.341

**Published:** 2017-09-28

**Authors:** Cosmos Yarfi, Evans Y.K. Ashigbi, Emmanuel K. Nakua

**Affiliations:** 1 Department of Physiotherapy and Rehabilitation Sciences, School of Allied Health Sciences, University of Health and Allied Sciences, Ghana; 2 Department of Population, Family and Reproductive Health, School of Medical Sciences, Kwame Nkrumah University of Science and Technology, Ghana

## Abstract

**Background:**

Accessibility implies making public places accessible to every individual, irrespective of his or her disability or special need, ensuring the integration of the wheelchair user into the society and thereby granting them the capability of participating in activities of daily living and ensuring equality in daily life.

**Objective:**

This study was carried out to assess the accessibility of the physical infrastructures (public buildings) in the Kumasi metropolis to wheelchairs after the passage of the *Ghanaian Disability Law* (Act 716, 2006).

**Methods:**

Eighty-four public buildings housing education facilities, health facilities, ministries, departments and agencies, sports and recreation, religious groups and banks were assessed. The routes, entrances, height of steps, grade of ramps, sinks, entrance to washrooms, toilets, urinals, automated teller machines and tellers’ counters were measured and computed.

**Results:**

Out of a total of 84 buildings assessed, only 34 (40.5%) of the buildings, 52.3% of the entrances and 87.4% of the routes of the buildings were accessible to wheelchair users. A total of 25% (13 out of 52) of the public buildings with more than one floor were fitted with elevators to connect the different levels of floors.

**Conclusion:**

The results of this study show that public buildings in the Kumasi metropolis are not wheelchair accessible. An important observation made during this study was that there is an intention to improve accessibility when buildings are being constructed or renovated, but there are no laid down guidelines as how to make the buildings accessible for wheelchair users.

## Introduction

Disability is a functional or structural limitation within the individual caused by physical, mental or sensory impairment (Driedger 1991). There are various groups of disabilities: the visually impaired, hearing impaired, cognitive impaired, speech disorders, intellectual and physical disabilities. Disability is defined as any restriction or lack (resulting from an impairment) of ability to perform an activity in the manner or within the range considered normal for humanity (WHO 1980). Thus, disability represents disturbance at the level of the person. In 1980, the World Health Organization classified disability using the International Classification of Impairment, Disabilities and Handicaps framework. The framework was revised in 2001 using the International Classification of Functioning to include three main components: body functions and structure, activities and participation and environmental factors (Useh, Moyo & Munyonga 2001; WHO 2001).

The definition of International Classification of Functioning is neutral to aetiology and emphasises function rather than the condition or disease. It also recognises the role of physical, social and environmental factors in affecting the outcome of disability. Thus, the environmental barriers that are deliberately erected by ‘abled-bodied members’ of the society compound the plight of people with disabilities (PWD) as far as participation and inclusion into the mainstream are concerned. The social model sees ‘disability’ as the result of the interaction between people living with impairments and an environment filled with physical, attitudinal, communication and social barriers (Oliver 2013). It therefore carries the implication that the physical, attitudinal, communication and social environment must change to enable people living with impairments to participate in society on an equal basis with others. A social model perspective does not deny the reality of impairment nor its impact on the individual. However, it does challenge the physical, attitudinal, communication and social environment to accommodate impairment as an expected incident of human diversity.

The social model seeks to change society to accommodate people living with impairment; it does not seek to change persons with impairment to accommodate society. It supports the view that PWD have a right to fully participate as citizens on an equal basis with other members of the society (Shakespeare 2006).

The medical model of disability also affects the way disabled people think about themselves. Society has for many years tended to treat PWD with pity and charity rather than as equals, entitled to the same comforts and benefits that society offer able-bodied citizens (Healey 2005). When policymakers and managers think about disability in an individual way, they tend to concentrate their efforts on ‘compensating’ people with impairments for what is ‘wrong’ with their bodies by, for example, targeting ‘special’ benefits at them and providing segregated ‘special’ services for them (Moyne 2012). Many PWD internalise the negative message that all their problems stem from not having ‘normal’ bodies. PWD too can be led to believe that their impairments automatically prevent them from participating in social activities (Moyne 2012). This attitude can make PWD less likely to challenge their exclusion from mainstream society. PWD inability to join in society is seen as a direct result of having impairment and not as the result of features of our society which can be changed. PWD do not want to be marginalised and shut out from the rest of society; instead, they want to be included where possible and given the same opportunities as everybody else to live and work as independently as possible.

PWD are entitled to the same rights as all other human beings and to equal opportunities in the society in which they live. Full participation means to take part in the social life and development of the communities in which they live (Perese 2013). According to Perese (2013), PWD have been stigmatised and victimised by prejudice, preventing them from assuming their rightful places in society. The rights and needs of PWD must therefore be considered in all spheres of planning and development of any nation. This means that everything must be done to eliminate physical or social barriers which prevent their full participation. PWD need an accessible physical and social environment with ramps and low height of steps to ambulate; therefore, an inaccessible environment is a major barrier that affects the ability to function as individuals and as members of their society. As a result of this, millions of children and adults in all parts of the world often face a life that is segregated and debased (Enable UN 1982). Therefore, the physical environment should be designed and equipped to meet the needs of a wide range of the population and supports equality and full participation of every member of the society. The government, civil society organisations, the community and the organisations of PWD have the responsibility to help improve accessibility of wheelchairs to public buildings. When the government, civil society organisations and the community are to implement changes in accessibility of the built environment, PWD must contribute to its implementation (Iwarsson & Ståhl 2003), because they know best the barriers they face and can offer practical solutions (Liverpool Independent/Integrated Living Project 1999; Lomas 1998).

It is estimated that about 650 million people in the world live with disabilities, representing 10% of the world’s population. Eighty per cent of PWD live in the developing regions: Africa, Asia, Latin America and the Caribbean (Driedger 1991). Iezzoni et al. (2001) estimated that 19 million people had some mobility difficulty, and the rates of associated problems of mobility disability are higher among women (11.8%) than men (8.8%). Approximately one out of every five Americans has a disability (McNeil 1999) with the disability likely to be higher in the older population (Raina et al. 1998). In Scotland, approximately one in every seven people has a disability, and 70% of PWD are aged over 65 years (Scottish Disability Rights Commission 2002). There are about 6 million people with disability in the UK (McGough 1994), and it has been estimated that in Great Britain as a whole, around one in four households contain at least one person with some form of disability and that around 70% of the PWD have some mobility difficulties (Cobbold 1997).

In Africa, an estimated 60–80 million people are living with disabilities, representing about 40% of the continent’s population (Driedger 1991).

In Ghana, the Statistical Service (2012) estimates the disability rate as 3% of the population. The three most prevalent types of disability in Ghana include visual impairment, hearing impairment and physical disabilities (Kuyini, Alhassan & Mahama 2011).

## Methods

### Study setting

The study was carried out in the Kumasi metropolis in the Ashanti region of the Republic of Ghana. The Ashanti region is the third largest of 10 administrative regions in Ghana, occupying a total land surface of 24 389 square kilometres or 10.2% of the total land area of Ghana. In terms of population, however, it is the most populated region with a population of 4 780 380 according to the 2010 Population and Housing Census in Ghana, accounting for 19.4% of Ghana’s total population.

Kumasi, the metropolitan capital, is the second most populous and largest cosmopolitan city of Ghana, the gateway to West Africa, with a population of 1 170 270, accounting for almost a third of the region’s population. The major land uses that make up the metropolis are residential, commercial, industrial, educational, civic and culture, open spaces and circulation.

Currently, the population of Kumasi is growing at an increasing rate with a growth rate of 5.47%, which is higher than the regional and national rates, and this stems from its vibrant commercial activities. The high rate of migration has also led to the emergence and construction of huge edifices and infrastructure, which raises the question of accessibility needs. The unique centrality of the city as a traversing point from all parts of the country makes it a special place for many to migrate to and a focal point for the establishment of companies and institutional infrastructure. The metropolitan assembly with its capital which also doubles as the regional capital has modern state of the art infrastructures housing second cycle institutions and institutions of higher learning which all have an accessibility concern for this study.

### Research design

The study was a descriptive cross-sectional survey to assess the accessibility of wheelchairs to public buildings in the Kumasi metropolis of the Ashanti Region, Ghana. A cross-sectional design was used because no hypothesis was stated, and the purpose of the study was descriptive in nature. It is a follow-up study with a sample of public buildings in the metropolis taken in order to make a representation of what happens to wheelchair accessibility in the metropolis.

The study was conducted from September 2010 to February 2011. The abridged form of Americans with Disabilities Act Accessibility Guidelines (ADAAG 1990) instrument was adopted as a tool for the data collection. There was no standard reference guideline for determining accessibility of buildings to wheelchair users in this environment, hence the use of the amended ADAAG on building. The ADAAG was adopted because it is the best international exemplar of best practices as far as accessibility for PWD is concerned.

### Sampling

#### Sample size

A total of 84 buildings were assessed: educational facilities, health facilities, ministries, departments and agencies (MDAs), sport and recreational buildings, religious institutions and banks due to on the vital role they play in society.

#### Sampling procedures

Different sampling procedures were applied in selecting the different buildings or institutions for the study. The second cycle institutions, religious facilities and banks were randomly selected. This was done by placing the names of all the buildings involved on a folded paper in a bowl and picking them one after the other until the required number was reached. But buildings from sports and recreational facilities, tertiary and health institutions as well as MDAs were purposively selected.

### Data collection

Twenty-one managers of selected buildings were contacted for approval and consent to carry out the study at their facility after the study was explained to them. The managers and custodians of these buildings answered structured questionnaires on how accessible their buildings were their awareness level of the Disability Law and the guidelines they have put in place to make the buildings accessible to PWD. Permission and consent to take the required measurements was also obtained from the appropriate authorities in charge of the buildings to be studied.

The data were collected mainly by direct observation of the buildings for the presence of accessible ramps, elevators, routes and entrances. Measurements of the routes, entrances, height of ramps and steps, height of sinks, urinals, water closets and toilets, automated teller machines (ATMs) and tellers’ counters were measured using tape measure. All entrances and connecting routes of buildings with multiple entry points were measured as well as some interior components of buildings in relation to accessibility. Priority was given to public buildings that were constructed after the passage of the *Disability Law in Ghana* (2006), to learn whether owners and occupiers of public buildings are taking steps to make their premises wheelchair accessible.

The total time it took for the measurements to be taken was 45 min.

The following measurements were taken and recorded to the nearest 0.1 centimetre:

- The entrance: the horizontal distance across the doorframe.
- The route: the horizontal distance between the edges of a corridor, passage and passageway.
- Parking area: the presence or absence of reserved parking spaces for wheelchair users in public buildings.
- Elevators: the presence or absence of elevators in buildings for wheelchair usage.
- Height of steps: the vertical distance from the bottom to the top of a step or any elevated surface located along the route of entry.
- Water closets and toilets: the presence or absence of water closets friendly for wheelchair users.
- Sinks: the presence or absence of sinks usable by wheelchair users.
- Public telephones: the presence or absence of telephones in public buildings.
- Urinals: the presence or absence of urinals usable by wheelchair users.
- ATMs: the presence or absence of an ATM that can be used by a person in a wheelchair.
- Height of counters for tellers in banking halls: distance from the floor to the top of a tellers’ counter.
- Height of ramp: the vertical distance from the bottom to the top of the ramp at the highest point.
- Length of ramp: the distance between the beginning and the end of the base of a ramp.
- The grade of ramp is deduced by finding the ratio of the height and the length of the ramp, that is:

$$Grade\;of\;ramp=\frac{Height\;of\;remp}{Length\;of\;ramp}$$

Accessibility, or otherwise, of each building was determined by comparing the measurements taken with the required dimensions as highlighted by an abridged form of the ADAAG.

A building’s exterior accessibility was determined when at least an entrance and its linking route(s) were found accessible, and interior accessibility was determined if it conforms to the guidelines of the instrument used for the study (Table 1).

**TABLE 1:** Summary of required dimensions for wheelchair accessibility using an abridged form of the Americans with Disabilities Act Accessibility Guidelines.

Parameters	Accessibility required	Remarks
Entrance	Minimum of 32 inches (81.5 cm)	Inaccessible if less than 81.5 cm
Route	Minimum of 36 inches (91.5 cm)	Inaccessible if less than 91.5 cm
Parking area	Minimum of 96 inches by 60 inches (244 cm by 152.5 cm)	Inaccessible if less than 244 cm by 152.5 cm
Elevators	Minimum area of 47 inches by 69 inches (119.5 cm by 175.5 cm)	Inaccessible if less than 119.5 cm by 175.5 cm
Height of steps	Maximum of 0.5 inches (1.3 cm)	Inaccessible if above 1.3 cm without being levelled or provision of a ramp
Water closets or toilets	Maximum height of wheelchair between 17 inches to 19 inches (43 cm to 48.5 cm)	Inaccessible if wheelchair height is above 48.5 cm
Sinks	Maximum height of 34 inches (86.5 cm) and depth of 6.5 inches (16.5 cm)	Inaccessible if height is above 16.5 cm
Public telephones	A clear ground space of at least 30 inches by 48 inches free ( 76 cm by 122 cm)	Inaccessible if ground space is less than 76 cm by 122 cm
Urinals	Either stall type or wall hung with elongated rim having a height of 17 inches (43 cm) from the floor	Inaccessible if height is above 43 cm
Automated teller machines (ATMs)	Maximum height from floor, 54 inches (137 cm) and reach depth, to button of 10 inches (25.5 cm)	Inaccessible if height and depth are above 137 cm and 25.5 cm
Height of counters	Maximum height of 44 inches (112 cm)	Inaccessible if above 112 cm
Grade of ramp, that is, height/length	Maximum of 1:12 with slope length less than 90 cm	Inaccessible if steeper than 1:12 or if slope of 1:12 is longer than 90 cm

*Source*: Hamzat, T.K. & Dada, O.O., 2005, ‘Wheelchair accessibility of public buildings in Ibadan, Nigeria’, *Asia Pacific Disability and Rehabilitation Journal* 16, 125–134

#### Tools for data collection

Tape measure, pens, digital camera and a structured questionnaire.

### Data management and analysis

The measurements done on the public buildings and the pictures and videos recorded were kept under lock and key. The data collected were cleaned and later organised by entering it into Statistical Package for Social Sciences (SPSS) for Windows version 16.0.

Descriptive statistics of frequency tables and percentages were obtained to present the data. The data collected were kept in a safe cabinet for six months after the end of the research.

### Ethical considerations

Ethical approval for the study was obtained from the Committee on Human Research, Publications and Ethics of the Kwame Nkrumah University of Science and Technology, School of Medical Sciences. The managers of the public buildings were informed about the research and given consent forms to sign and confirm their readiness to be interviewed and allow measurements to be taken on their buildings. They were assured of the confidentiality of the information being sought.

## Results

A total of 84 public buildings that offered essential services such as education, health, MDAs, sports and recreation, religious groups (churches and mosques) and banks were assessed. The distribution of the buildings among the essential services showed that 12, 47, 6, 5, 10 and 4 buildings were studied under health, education, MDAs, sports and recreation, religious groups and banks, respectively.

The results further showed that 446, 1236, 70, 149, 107 and 9 entrances of buildings were assessed under health, education, MDAs, sports and recreation, religious groups and banks, respectively. Table 2 shows that 236, 29, 25, 125, 39 and 7 routes were also studied under health, education, MDAs, sports and recreation, religious groups and banks, respectively.

**TABLE 2:** Distribution of buildings, entrances, and routes accessible to wheelchair users.

Types of buildings	Buildings		Entrances		Routes	
	Accessible *N* (%)	Inaccessible *N* (%)	Accessible *N* (%)	Inaccessible *N* (%)	Accessible *N* (%)	Inaccessible *N* (%)
Health (*N* = 12)	11 (91.7)	1 (8.3)	355 (79.6)	91 (20.4)	28 (96.6)	1 (3.4)
Education (*N* = 47)	16 (34.0)	31 (66.0)	484 (39.2)	752 (60.8)	236 (100)	0 (0.0)
MDAs (*N* = 6)	0 (0.0)	6 (100.0)	15 (21.4)	55 (78.6)	25 (100)	0 (0.0)
Social/recreation (*N* = 5)	2 (40.0)	3 (60.0)	102 (68.5)	47 (31.5)	69 (55.2)	56 (44.8)
Religious groups (*N* = 10)	2 (20.0)	8 (80.0)	89 (83.2)	18 (16.8)	39 (100)	0 (0.0)
Banks (*N* = 4)	3 (75.0)	1 (25.0)	9 (100.0)	0 (0.0)	6 (85.7)	1 (14.3)

**Total = 84**	**34 (40.5)**	**50 (59.5)**	**1054 (52.3)**	**963 (0.0)**	**403 (87.4)**	**58 (0.0)**

*Source*: Authors’ own work

A total of 11 (91.7%) out of 12 health, 16 (34%) out of 47 education, 2 (40%) out of 5 sports and recreation, 2 (20%) out of 10 religious and 3 (75%) out of 4 bank buildings were wheelchair accessible. None of the six buildings under MDAs had wheelchair access. For the entrances of the buildings, 355 (79.6%) out of 446 health, 484 (39.2%) out of 1236 education, 15 (21.4%) out of 70 MDAs, 102 (68.5%) out of 149 sports and recreation and 89 (83.2%) out of 107 religious buildings were wheelchair accessible. All nine (100%) entrances of banking halls were wheelchair accessible. The routes of buildings assessed recorded a 28 (96.6%) out of 29 health, 69 (55.2%) out of 125 sports and recreation and 6 (85.7%) out of 7 bank buildings were accessible to wheelchair users, whereas the routes in the education, MDA and religious buildings recorded 100% accessibility to wheelchair users. In all, a total of 40.5%, 52.3% and 87.4% of buildings, entrances and routes, respectively, were wheelchair accessible as shown in Table 2.

A total of 52 public buildings representing 61.9% of the 84 buildings assessed had more than one floor. Out of these 52 buildings, only 13 of them representing 25% had an elevator to connect the different levels of the buildings. The remaining 39 representing 75% were not fitted with elevators indicating that wheelchair users cannot access the other floors of the buildings. All 8 health buildings assessed with more than one floor had elevators for wheelchair access, and only 5 out of the 33 educational buildings were fitted with elevators. But none of the 11 MDA, religious and sports and recreational buildings had elevators for wheelchair access as shown in Table 3.

**TABLE 3:** Distribution of buildings with more than one storey fitted with elevators.

Type of building	Elevators *N* (%)	No elevators *N* (%)
Health (8)	8 (100.0)	-
Education (33)	5 (15.2)	28 (84.8)
MDAs (5)	-	5 (100.0)
Social/recreation (1)	-	1 (100.0)
Religious groups (5)	-	5 (100.0)
Banks (0)	-	-

**Total (52)**	**13 (25.0)**	**39 (75.0)**

*Source*: Authors’ own work

In all, a total of 137 entrances to washrooms representing 22.1%, 158 toilets and water closets representing 29.1%, 222 sinks representing 45%, 5 urinals representing 3% and 3 tellers’ counters representing 75% were accessible to wheelchair users. All the ATMs were accessible to wheelchair users as shown in Table 4.

**TABLE 4:** Distribution of washrooms, toilets, sinks, urinals, automated teller machines and tellers’ counters accessible to wheelchair users.

Parameters	Accessible *N* (%)	Inaccessible *N* (%)	Total
Washroom entrance	137 (22.1)	482 (77.9)	619
Toilets and water closets	158 (29.1)	385 (70.9)	543
Sinks	222 (45.0)	271 (55.0)	493
Urinals	5 (3.0)	161 (97.0)	166
ATMs	4 (100.0)	-	4
Tellers’ counters	3 (75.0)	1 (25.0)	4

*Source*: Authors’ own work

A total of 21 managers and custodians of public buildings were interviewed on the accessibility concerns of their buildings. Fourteen of the managers had plans to provide ramps when renovating their facility. Only 6 of the managers of institutions have a written document to make their buildings wheelchair friendly, whereas 16 of the managers said they had plans to construct buildings that are wheelchair friendly. A total of 18 of the managers were aware of the *Disability Act*, whereas 10 of them were aware of sections 6 and 7 of the Act. A total of 13 of the managers said they have wheelchair users visiting their facility.

## Discussion

The results showed that averagely one-third, that is, 34 out of the 84 buildings assessed, were accessible to wheelchair users. This study showed that physically challenged persons in Kumasi who use wheelchairs to ambulate can only gain access to 40.5% of the public buildings which house facilities that provide services for health, education, MDAs, sports and recreation, religious and banking needs. This study contradicts a similar study by Hamzat and Dada (2005), who reported just 20% of accessible public buildings to wheelchair users in Ibadan, Nigeria. The higher percentage of accessible buildings recorded in this study unlike the one reported by Hamzat and Dada could be attributable to the larger number of buildings (84) assessed in this study compared to the 38 buildings surveyed in the study by Hamzat and Dada. It could also be because of the awareness of disability issues by PWD themselves and their organised groups after the passage of the Disability Law in Ghana, whereas in Nigeria, there were no legislations governing disability issues at the time of the study.

The results of the structural domains evaluated revealed that 52.3% of the entrances of public buildings were accessible to wheelchair users. This result contradicts a study by Useh et al. (2001), who reported 71% accessibility for entrances into public buildings in the central business district of Harare, Zimbabwe, but agrees with a study by Hamzat and Dada (2005), who reported 50% entrance accessibility after a study to determine the wheelchair accessibility of public buildings in Ibadan, Nigeria. The low level of entrance accessibility recorded in this study as compared to the study by Useh et al. could be accounted for by the high steps and thresholds along the entrances. These steps and thresholds could have been easily removed or levelled to ensure wheelchair access to these entrances. An alternative way of enhancing entrance accessibility is to provide ramps with grade not more than 1/12 as suggested in the ADAAG (1990) and widening the width of doorways.

The study also recorded 87.4% of routes of public buildings accessible to wheelchair users. The results seen contradict a study by Figoni et al. (1998) who reported 48% route accessibility and Cardinal and Spaziani (2003) who reported 58% route accessibility of physical fitness facilities. The fact that a higher percentage of the routes were within the required dimensions for wheelchair accessibility might have been coincidental, and not really meant to meet the needs of wheelchair users. Because a large number of people make use of these public places, there was the need for wider routes to allow easy movement of human traffic before, during and after programmes organised in these public buildings.

Among the 52 buildings with more than one floor assessed, only 13 of the buildings representing 25% had an elevator to connect the different levels of floors. All 8 health buildings assessed were accessible to wheelchair users, whereas only 5 out of the 33 education buildings with more than one floor were fitted with an elevator but 2 of the elevators were not functioning. None of the buildings assessed under sports and recreation, MDAs and religious groups with more than one floor were fitted with an elevator to connect the different floors. For the remaining 39 buildings, the wheelchair users would only be able to access the ground floors, and in some instances, these too were inaccessible because of high height of steps and threshold. This is in contradiction to the 83% accessibility of elevators in a study by Useh et al. (2001) to evaluate accessibility of wheelchairs into public buildings in the central business district of Harare, Zimbabwe. The difference in these two particular results may be because of the fact that Useh et al. assessed only 20 buildings as compared to the 84 buildings assessed in this study. Moreover, in Ghana, it is only obligatory for owners and occupiers of public buildings with more than four floors to provide elevators in their buildings, and this could have contributed to the low level of buildings with more than one floor fitted with elevators seen in this study.

A total of 11 out of 12 buildings in the health sector representing 91.7% assessed were found to be wheelchair accessible. Similarly, Rimmer et al. (2005) also reported an appreciable level of accessibility (58.5%) of health facilities in 9 out of the 10 geographic regions in the United States. The higher level of accessibility in this study could be because of the fact that architects and construction engineers took into account the needs of patients visiting the hospitals that have mobility challenges and thus use wheelchairs for ambulation and need accessible ramps and facilities to easily manoeuvre their way inside the buildings. It could also be attributed to the fact of awareness creation since the passage of the Disability Law on the special needs of PWD in society.

The low accessibility of buildings for educational purposes (34%) is suggestive of inappropriate architectural designs of such buildings. It appears that the major efforts of the owners were devoted to making these architectural structures masterpieces, with little or no consideration for individuals who are wheelchair-mobile. This could be a reason why PWD, in particular the wheelchair users, are not encouraged to be educated because of the obstacles they face in accessing buildings in the educational sector (Christensen, Blair & Holt 2007). Two out of the four (50%) school libraries measured in educational buildings were accessible. This implies that wheelchair-mobile students would have a 50% access chance to the libraries in the educational sector. This study contrasts a similar report by Hamzat and Dada (2005), who observed a low level of accessibility of buildings used for educational purposes (6.7%). This could be because of the fact that a higher number of buildings were assessed under the educational buildings (47) as opposed to the three buildings assessed by Hamzat and Dada’s study.

The 0% accessibility of six buildings measured under MDAs housing social welfare, immigration service, regional directorate of Ghana Health Service, labour office and national vocational training institute implies that wheelchair-mobile citizens would not be able to access the services being provided by these government functionaries. This also means that wheelchair users would be hindered from visiting the buildings that serve the purposes of employment, social welfare, acquisition of passports and those seeking vocational training. It also implies that services provided by these departments would only probably reach the wheelchair users by indirect means, as these individuals would not be able to get to the location where these services are provided while in their wheelchairs. The alternative would be for such persons to be carried into these buildings; this act has a potentially negative psychological effect on the individual (Pierce 1998).

The sports and recreation buildings recorded 40% accessibility. One of the national sports stadia in the metropolis assessed accounted for the high incidence of accessibility under the sports and recreational centres. The sports stadium was constructed to suit the regulations of world football governing body, Federation of International Football Associations (FIFA), on accessibility. The implication of the low level of wheelchair accessibility recorded for the sports and recreational buildings is that wheelchair users would not be able to fully participate in all social and recreational activities which serve the purposes of relaxation, recreation and health promotion. This therefore denies PWD access to sport and recreational facilities and is a violation of Article 30 of the United Nations Convention on the Rights of PWD (UNCRPD) which provides for the participation of PWD in cultural life, recreation, leisure and sport. Section 5(b) of Article 30 specifically obliges state parties to take appropriate measures:

‘to ensure that ‘PWDs have an opportunity to organize, develop and participate in disability-specific sporting and recreational activities and, to this end, encourage the provision, on an equal basis with others, of appropriate instruction, training and resources.’ (UNCRPD 2006:23)

The benefits of outdoor recreation experiences are largely the same for people with and without disabilities (McAvoy & Lais 1999).

The low level of accessibility recorded under the religious buildings (20%) is indicative that the wheelchair-bound individual is limited or prevented from participating in most religious activities and worshipping his or her maker. None of the five buildings assessed under mosques were wheelchair accessible, whereas only two of the five church buildings were accessible to wheelchair users. The accessible church buildings were probably just a matter of coincidence and not a conscious effort of making them accessible. This low level of accessibility could be attributable to the religious view of disability in society that views PWD as having committed a wrong doing and that disability is the punishment for the offence committed (Zedda 2008). The low level of accessibility of religious buildings to wheelchair users is also a violation of their freedom of religion as provided for in Article 21(1)(c) of the 1992 Ghanaian Constitution.

The buildings under the banking sector recorded an appreciable level of accessibility (75%). All the three private banks assessed were found to be accessible, whereas the only government bank was inaccessible to wheelchair users. The private banks were made accessible by renovating them to incorporate ramps that can easily be used by a wheelchair user. This could be because of the fact that the private banks are making their premises accessible to attract a large customer base and not because of meeting the special needs of the wheelchair user, for the ramp gradient does not follow the principles of universal design. The inaccessibility of the government bank buildings could be because of the lack of funds from central government for renovation purposes and the bureaucratic procedures to follow for renovations to be done on the buildings.

Wheelchair accessibility of entrance to washrooms, toilets, sinks and urinals were 22.6%, 26.4%, 52.9% and 5.6%, respectively, in new public buildings, whereas the entrance to washrooms, toilets and sinks of old public buildings recorded a 21.9%, 30.8% and 41.4% accessibility, respectively. The 26.4% and 30.8% toilet accessibility recorded in this study contradicts a similar study by Useh et al. (2001) who reported 51% of toilet accessibility to wheelchair users into public buildings in the central business district of Harare, Zimbabwe. This study further showed that a wheelchair user will experience many difficulties entering the washrooms in most of the buildings assessed, let alone have access to the toilet facilities in the buildings. It was only some of the buildings under the health sector and Baba Yara Sports Stadium that had special toilet facilities for use by the wheelchair users. This could be because of the fact that the sports stadium needed to meet the world governing body (FIFA) regulations on accessibility needs and it was not to meet the needs of the wheelchair users. None of the urinals in the buildings assessed were accessible to the wheelchair user.

In the banking sector, all the four ATMs assessed were accessible (100%) to the wheelchair user, whereas 75% of the tellers’ counters were accessible to the wheelchair user. All the three private banks assessed were wheelchair accessible, while the only government bank assessed was inaccessible. There was an intention to make bank buildings wheelchair accessible by creating ramps during renovations of these banks. The provision of accessible links to the buildings in the banking sector may be a way of increasing their customer base to attract all manner of people to their premises and not to create an accessible environment for the wheelchair user.

### Limitations of the study

- There was no standard reference guideline for determining accessibility of buildings to wheelchair users in this environment, hence the use of the amended ADAAG.
- There were unequal numbers of buildings assessed in the five essential services of the study because of inadequate buildings in some of the institutions.
- Smaller sample size of 84 public buildings.

### Recommendations

- There should be the development of a national building code using the principles of universal design to ensure all public buildings are accessible to all persons.
- There is the need for awareness raising around issues on removing and breaking physical barriers in society and provision of an accessible physical environment for every member of the society. This should be the responsibility of every citizen in the country, and the Ghana Federation of the Disabled should team up with state institutions responsible for dissemination of information to reach a large proportion of the populace.
- There should be liaison between the end users (PWD) and various professionals (politicians, engineers, lawyers, architects, physiotherapists, disability management practitioners and occupational therapists) during the development of a framework for the construction of buildings in order to make them accessible to wheelchair users in Ghana. This is because the end users know best the barriers they face and therefore will make meaningful contributions to the framework. The findings of this study indicate a great challenge to the aforementioned professionals.
- The National Council for PWD that has an oversight responsibility on disability issues in Ghana should liaise with the association of architects and contractors on the need and how to design and construct accessible public buildings in Ghana.
- There is the need to set up a regulatory body that will have an oversight responsibility of all public buildings constructed in the country to make sure they are wheelchair accessible before sanction or approval is given for final construction.
- More attention should be paid in providing accessibility links during construction and renovation of public buildings, especially those of education, MDAs, religious and sports or recreation buildings in Kumasi and across the country.

## Conclusions

An important observation made during this study was that there is an intention to improve accessibility when buildings are being constructed or renovated. This study described how accessible public buildings should be to wheelchair users because the country has a Disability Law but lacks the requisite guidelines (legislative instrument and building codes) on how to make public buildings accessible to all. It was also realised that narrow entrances and routes of buildings, raised steps at entrances of the buildings and steep ramps rendered most of the buildings inaccessible to wheelchair users.

There is the need for the Ghanaian government to develop building codes and guidelines using universal design principles to ensure all public buildings are wheelchair accessible. This can be achieved by liaison between wheelchair users and various professionals (disability management practitioners, occupational therapists, physiotherapists, engineers and architects).

It is important to increase the level of wheelchair accessibility to public buildings; this will facilitate independence, integration and reintegration of wheelchair users into the society. It will also ensure equity for all and thereby contribute to the achievement of the sustainable development goals in Ghana.

## Significance of the study

The development of every nation evolves with the housing of its agencies, organisations and institutions to serve the general public. A building not accessible by persons with physical disability means they have been excluded as far as usage of the facility is concerned. In almost all societies of the world, major obstacles continue to hamper the development of PWD, thus preventing them from exercising their rights and freedom, making it especially difficult for them to participate fully in the activities of their societies.

The results of this study will reflect the trend of accessibility of public buildings to wheelchair users in Kumasi. The outcome of this research will be used as part of an advocacy for the enforcement of policy to guide appropriate MDAs to ensure accessibility of self-propelling wheelchair users to all public places or buildings in the country. It is very important to look at the abilities, not the disabilities, of PWD, and create accessible environments for them to contribute their quota to national development and integrate the wheelchair user into the society by providing access to buildings.

This study will help address the inadequacies of the Disability Law (2006) which talks about owners or occupiers of a place to which the public has access needing to provide appropriate facilities that make the place accessible to and available for use by a PWD without providing accessibility guidelines for owners and occupiers of such buildings to follow. This will ensure that all persons have the opportunity to secure suitable employment, participate in social and recreational activities, have access to healthcare services and acquire formal education.
